# Broad Diversity of Fungi in Hospital Water

**DOI:** 10.1155/2020/9358542

**Published:** 2020-07-04

**Authors:** Máira Gazzola Arroyo, Adriano Menis Ferreira, Oleci Pereira Frota, Natalia Seron Brizzotti-Mazuchi, Jacqueline Tanury Macruz Peresi, Marcelo Alessandro Rigotti, Carlos Eduardo Macedo, Alvaro Francisco Lopes de Sousa, Denise de Andrade, Margarete Teresa Gottardo de Almeida

**Affiliations:** ^1^Postgraduate Program in Microbiology, São Paulo State University, Sreet Cristóvão Colombo, 2265 Garden Nazareth, São José do Rio Preto, SP 15054-000, Brazil; ^2^Postgraduate Program Nursing and Medical Course, Federal University of Mato Grosso do Sul, Três Lagoas, MS 79600-080, Brazil; ^3^Postgraduate Program in Nursing Course, Federal University of Mato Grosso do Sul, Campo Grande, MS 79070-900, Brazil; ^4^Department of Infectious and Parasitic Diseases, School of Medicine of São José Do Rio Preto, São José do Rio Preto, SP 15090-000, Brazil; ^5^Adolfo Lutz Institute, Regional Laboratory of São José do Rio Preto, São José do Rio Preto, SP 15060-020, Brazil; ^6^Nursing Course, Federal University of Mato Grosso do Sul, Três Lagoas, MS 79600-080, Brazil; ^7^Postgraduate Program in Health and Development in the Midwest, Federal University of Mato Grosso do Sul, Três Lagoas, MS 79600-080, Brazil; ^8^Department of General and Specialized Nursing, University of São Paulo, Ribeirão Preto School of Nursing, Ribeirão Preto, SP 14049-900, Brazil

## Abstract

**Introduction:**

Some studies have reported the occurrence of microorganisms isolated from water. Considering these microorganisms, fungi are known to occur ubiquitously in the environment, including water, and some are pathogenic and may cause health problems, especially in immunocompromised individuals. The aim of this study was to identify fungi in hospital water samples and to correlate their presence with the concentration of free residual chlorine.

**Methods:**

Water samples (100 mL) were collected from taps (*n* = 74) and water purifiers (*n* = 14) in different locations in a university hospital. Samples were filtered through a nitrocellulose membrane and placed on Sabouraud dextrose agar and incubated for 24 hours at 30°C. Fungi were identified according to established methods based on macroscopic and microscopic characteristics (filamentous) and physiological tests (yeasts). Free chlorine residual content was measured at the time of sample collection.

**Results:**

Seventy species of fungi were identified in the water samples and about 56% of the water samples contained culturable fungi. *Cladosporium oxysporum*, *Penicillium spinulosum*, and *Aspergillus fumigatus* were the most common filamentous fungi. *Aureobasidium pullulans* and *Candida parapsilosis* were the most common yeasts. Chemical analyses revealed that free residual chlorine was present in 81.8% of the samples within recommended concentrations. Among samples from water purifiers, 92.9% showed low levels of free residual chlorine (<0.2 mg/L). There was no significant association between chlorine concentrations (either within or outside the recommended range) and the presence of filamentous fungi and yeasts.

**Conclusions:**

This study showed that hospital water can be a reservoir for fungi, some of which are potentially harmful to immunocompromised patients. Free residual chlorine was ineffective in some samples.

## 1. Introduction

The guarantee of human health is related to several factors, including water quality. To be in ideal conditions for consumption, water must obey microbiological standards, which work as indicators of contamination [1, 2]. Some studies have reported the occurrence of microorganisms isolated from different sources of water, indicating potential risks to human health, especially when occurring in the hospital environment, whose patients are immunocompromised, and making them susceptible to other diseases [3–8].

In this context, fungi are known to occur ubiquitously in the environment and some studies on hospital water samples have revealed their presence [3, 4]. These, when found in water for human consumption, can change the taste and smell, which makes its aspect becomes unpleasant [9, 10].

Besides, some fungi are considered pathogenic and cause several impacts on human health, such as allergies, respiratory illness, life-threatening meningitis, mycoses, and invasive and contagious infections [8, 11].

Others are capable of producing mycotoxins and other secondary metabolites that are toxic, causing respiratory problems, and can also be carcinogenic, reducing the immunity, mainly of immunocompromised patients, becoming a threat to human health [7, 10, 12, 13].

In addition to microbiological parameters, chemical parameters must also be monitored, in order to control waterborne diseases. There are several disinfectant agents, but in general, chlorine is the main product used for water disinfection, because it is the most effective agent against the growth of pathogenic organisms [2, 14].

Chlorine is a substance used in the water supply system to oxidize organic matter from springs that can appear in the distribution system [2, 14]. However, its consumption can be harmful to health; thus, in households, water purifiers are used to remove suspended solids and chemical elements, such as chlorine, leaving the water clean and proper for consumption, in addition to avoiding any kind of odor and taste [15].

The Brazilian legislation that regulates the control and surveillance procedures of the quality of water for human consumption only contemplates potability in relation to bacteriological parameters. [1]. In this sense, there is currently no specific legislation for fungi in water, as many of them can cause several diseases as demonstrated above, especially in immunocompromised individuals. Researches involving drinking water quality, including fungi, are essential for human health. Thus, the aim of this study was to identify the presence of fungi in hospital water and to correlate free residual chlorine concentrations with the presence of these microorganisms.

## 2. Materials and Methods

### 2.1. Sample Collection

Water samples (100 mL) were collected from taps (located in the bathrooms of the inpatient bedrooms) (*n* = 74) and water purifiers (*n* = 14) (located in the hospital corridors) in different floors in a university hospital in São José do Rio Preto, São Paulo, Brazil, between April and June 2012 (Table 1). Eight water samples were collected from each floor, except for the oncology department and intensive care, with 16 samples each.

### 2.2. Filtering of the Samples and Microbiological Analysis

Thus, after disinfection with 70% alcohol and a continuous streaming flow of water for two minutes, 100 ml of water was poured into Whirl-Pak® THIO bags with 1.8% sodium thiosulfate to neutralize the disinfecting action of chlorine. Immediately, the bags were transported in isothermal boxes to a microbiology laboratory as specified by the World Health Organization (WHO) and the American Public Health Association (APHA) [2, 16].

Each sample was filtered through a nitrocellulose membrane (47 mm diam., 0.45 *μ*m pore size), placed on a Petri dish with Sabouraud dextrose agar (containing chloramphenicol) in the presence of the Bunsen burner flame to maintain the sterile environment, and incubated for 24 hours at 30°C. Following that, the membrane was removed from the dish, which was kept under similar incubation conditions: 30°C for 15 days [16–18].

All morphologically distinct colonies were investigated; however, only one isolate was considered if identical strands were identified. Fungal isolates were identified according to established methods based on macroscopic and microscopic characteristics. Microculture was used to stimulate the development of fruiting bodies: a small piece of potato dextrose agar was placed on a sterilized slide on a sterilized Petri dish. Fungi from a recent subculture were grown in all four sides of the agar block, which was covered with a sterilized coverslip. Following that, 2 mL of sterile distilled water was added to the dish to prevent the desiccation of the growth medium. The dish was topped and incubated at room temperature for seven to ten days, until the development of hyphae was detected. After fungal growth, the coverslip was removed with tweezers and a drop of lactophenol cotton blue stain was added to the material, so the spores and hyphae could be visualized using an optical microscope [17].

Yeasts were analyzed based on their characteristics (the size and color of colonies) and on the results of physiological tests, such as the assimilation of different sources of carbon and nitrogen. Yeast was grown using the pour plate technique: 2 mL of a suspension of the colonies (distilled water plus colonies was used until reaching Mcfarland scale 7) was homogenized in each mixed medium (yeast nitrogen base and yeast carbon base) distributed into two Petri dishes. Small aliquots of different carbohydrates were added to the surface of the carbon-free medium (at different points on a single plate) to serve as carbon sources. Similarly, in another dish, nitrogen sources were placed on the surface of the nitrogen-free medium and incubated at 25°C for 24 to 48 hours. After this period, the results were read according to the formation of a growth halo, which indicated assimilation according to the carbon and/or nitrogen sources used. Thus, the sources used allowed for the identification through taxonomic identification keys according to Kurtzman and Feel [18].

### 2.3. Chlorine Concentration Analysis

Free chlorine residual content was measured at the time of sample collection. To determine the free chlorine residual content in the water, 10 mL of the sample and 0.5 mL NN diethyl-p-phenylenediamine (DPD) reagent were added to a cuvette (1). The mixture was stirred for homogenization and measured with an electronic colorimeter device (HI96711 C–Hanna Instruments). To meet the standards required by Ordinance 2.914/2011 [1], the free residual chlorine concentration of samples must range from 0.2 to 2 mg/L.

### 2.4. Statistical Analysis

The data analysis was performed with the software Minitab, version 17. Fisher's exact test was used to analyze the association between the presence of filamentous fungi and yeast, and chlorine levels (either within or outside the recommended range). The significance level was set at *p* ≤ 0.05.

## 3. Results

### 3.1. Presence of Fungi in Samples

Seventy species of fungi were identified in the water samples. In total, 55.6% (49/88) of samples were contaminated, of which 89.8% (44/49) were from taps and 10.2% (5/49) were from water purifiers. Filamentous fungi were more common than yeasts: 48 species were found in 47% (42/88) of water samples. *Cladosporium oxysporum* was the most common fungus and widely distributed across the hospital; followed by *Penicillium spinulosum*, which was also distributed across the floors; however, *Aspergillus fumigatus* was more common in the transplant unit.

Table 1 shows the fungi distribution according to the origin of the samples.

Table 2 shows the fungi species isolated in water from taps and purifiers.

Twenty-two yeast species were found in 17% (15/88) of water samples: the highest number of yeasts was found in the infectious diseases department and the oncology department. *Aureobasidium pullulans* and *Candida parapsilosis* were the most prevalent yeasts; the first was found in the postoperative room, oncology department, and pediatrics department, and the second, in half of the hospital's floors.

### 3.2. Presence of Chlorine in Samples

Chemical analyses revealed that 81.8% (72/88) of the samples presented free residual chlorine levels within the recommended parameters (0.2 to 2 mg/L). However, almost all water purifier samples (92.9%; 13/14) had concentrations below 0.2 mg/L. There was no significant association between chlorine parameters (either within or outside recommended levels) and the presence of filamentous fungi (*p*=0.12) and yeasts (*p*=0.10).

## 4. Discussion

The lack of limits of fungus quantification in water for human consumption was relevant to the development of this article, since pathogenic fungi are being isolated from different kinds of sources of water [3–8] and there is no legislation to control and monitor this occurrence, both in Brazil and in the world. In this study, most samples demonstrated fungal growth regardless of the location in the hospital (Table 1).

Many of these microorganisms are found in materials that are directly in contact with water, such as soil, wood, and decomposing materials, which explains their persistence in aquatic environments [19]. This environment, together with chemical-physical characteristics (turbidity, temperature, and pH) and organic compounds, is favorable to microbial growth, making these environments human health-threatening [18].

Another relevant point is that many elements that compose water distribution systems, such as iron, hydrogen, and phosphate compounds, favor microorganisms' growth [14]. These factors, together with the lack of maintenance of water distribution systems, contribute to an increased risk of microbial contamination. Therefore, monitoring those microorganisms is critical when assessing the integrity of distribution systems [1, 14].

In this study, filamentous fungi and yeasts were more common in tap water samples on all floors than in samples from purifiers (Table 1). Previous studies have shown similar results in hospital water and drinking water [3, 20]. In this study, these fungal groups, considered opportunistic pathogens, were common in different hospital sectors, which may pose a risk to immunocompromised patients, especially if the microorganisms are inhaled [21–23].

The high prevalence of *Cladosporium* spp*., Penicillium* spp., and *Aspergillus* spp. in this study has been reported in other studies on the water supply systems of laboratories and other locations [3, 20]. These fungi are considered dematiaceous fungi responsible for causing mycoses and other types of infections, such as cutaneous, subcutaneous, invasive, and contagious infections [11]. Furthermore, the genera are associated with the production of mycotoxin [10, 13, 24]. A study by Paterson et al. [5] found mycotoxins in cistern water and, if ingested, they may cause some health problems, such as vascular and some types of mycosis [25]. Aspergillosis caused by *Aspergillus* spp. is very common in immunocompromised patients and is the second most frequent cause of nosocomial fungal infections [26], a worrying datum when isolating this genus in the transplantation unit, as demonstrated in the present study.

Yeast groups have been isolated from different water sources such as rivers and lakes [27]. In this study, the high occurrence of yeast in the infectious diseases department and oncology department is concerning because of the debilitated immune systems of patients. Yeast species *Aureobasidium pullulans* and *Candida parapsilosis*, which were found in several locations in this study and in prior studies, are considered opportunistic pathogens [27, 28]. For example, *Candida* spp. is the fourth leading cause of nosocomial bloodstream infections in the United States [29]. When ingested by immunocompromised individuals, yeasts can travel through the gastrointestinal mucus and enter the bloodstream, spreading to other organs [29].

Although rare, molecular studies have shown that the fungal isolates from hospital waters presented the same genotypic characteristics as isolated fungi obtained from patients, which indicates that these water reservoirs are a source of infections in patients, especially immunocompromised ones [4, 6, 10, 30]. Ingestion, inhalation, contact with skin, and mucous membranes are some routes by which people can be exposed to fungi in drinking water [10].

In relation to the chlorine, in this study, free chlorine residual levels of most tap samples were within the recommended standards (Table 1). However, only one purifier water sample presented the 0.2 mg/L minimum (Table 1), because water purifiers function by removing chlorine from water to remove its flavor and odor, a mechanism that contributes to microorganism's growth in the end part of the system [15]. Notably, the periodical maintenance of purifiers was within the deadline.

However, we must emphasize that regardless of most tap water samples presenting adequate concentrations of free chlorine residuals, fungi were commonly present (all sampling points). Although chlorine is widely used to remove many harmful microorganisms in water, fungi were discovered to be more resistant to chlorine inactivation than bacteria [20].

Fungi produce secondary metabolites that can contribute to microbiological corrosion in water pipes. Consequently, changes in proper disinfection can occur and the concentrations of residual chlorine in the treated distribution water system can be altered [10, 30].

Moreover, chlorine-resistant fungal spores can also occur, thereby favoring the formation of biofilm in the inner surfaces of pipes, debris, or sediments in the drinking water distribution system, which favors cell survival even in the presence of chemical products [14, 31].

In the present study, biofilm formation may have occurred in the pipeline, since, in the infectious diseases, oncology and intensive care departments presented the highest fungal contamination of all floors.

Most of the fungal genera described in the study are dematiaceous fungi that are capable of secreting melanin in their cell walls, making them thick-walled species that can resist water treatment [6, 10]. Another study also demonstrated the resistance of *Cladosporium* spp., *Aspergillus* spp., and *Penicillium* spp. to chlorine in drinking water sources, so that they remain in the distribution system for long periods [20].

Yeasts can also be resistant to chlorine, which means that they can survive and accumulate in water distribution systems [10, 32]. In the present study, in the sample where there was no fungal growth, the chlorine may have inhibited the growth of those microorganisms, but, as previously demonstrated, one cannot state that the chlorine efficacy was proved, since the samples with chlorine also had no fungal growth.

## 5. Conclusions

In conclusion, hospital tap water and purified water can be a reservoir for fungi, some of which are opportunistic pathogens, which can put people's health at risk, especially in immunocompromised individuals. Free residual chlorine was ineffective in some samples, allowing for the growth of microorganisms. Therefore, the present study demonstrates that more researches should be developed aiming to contemplate the monitoring of fungi in several water sources.

## Figures and Tables

**Table 1:** Results of analysis stratified by the origin of the sample.

Hospital sector	Number of samples		Number of positive samples				Number of samples with free chlorine residual within the limits	
			Filamentous		Yeasts			
	*T*	*P*	*T*	*P*	*T*	*P*	*T*	*P*
Haemodialysis sector (ground floor)	7	1	2	0	1	0	7	1
Surgical centre (1^st^ floor)	6	2	4	1	0	0	6	0
Infectious diseases department (2^nd^ floor)	6	2	2	2	4	1	4	0
Oncology department (3^rd^ floor)	13	3	5	1	1	2	13	0
Pediatrics department (4^th^ floor)	7	1	4	0	2	0	7	0
Cardiology department (5^th^ floor)	6	2	2	0	1	0	6	0
Postoperative room (6^th^ floor)	6	2	5	0	1	0	6	0
Intensive care (7^th^ floor)	15	1	7	0	1	0	14	0
Transplant unit (8^th^ floor)	8	0	7	0	1	0	8	0
Total	74	14	38	4	12	3	71	1

*T*: tap water; *P*: purifiers.

**Table 2:** Fungi species isolated in hospital water from taps and purifiers.

	*n*	*T*	*P*	Hospital sector
Filamentous				
*Cladosporium oxysporum*	8	7	1	1^st^, 3^rd^, 5^th^, 6^th^, and 7^th^ floor
*Penicillium spinulosum*	5	4	1	Ground floor, 1^st^, and 3^rd^ floor
*Aspergillus fumigatus*	5	5	0	8^th^ floor
*Curvularia clavata*	3	3	0	4^th^ and 8^th^ floor
*Penicillium citrinum*	2	2	0	2^nd^ and 8^th^ floor
*Trichoderma koningii*	2	1	1	1^st^ and 2^nd^ floor
*Trichoderma harzianum*	2	1	1	7^th^ and 8^th^ floor
*Acremonium hyalinulum*	2	2	0	7^th^ floor
*Penicillium purpurogenum*	1	1	0	3^th^ floor
*Penicillium verruculosum*	1	1	0	2^th^ floor
*Cladosporium spaerospermum*	1	1	0	7^th^ floor
*Alternaria alternata*	1	0	1	2^nd^ floor
*Nigrospora sphaerica*	1	1	0	3^rd^ floor
*Scytalidium dimidiatum*	1	1	0	3^rd^ floor
*Fusarium sacchari*	1	1	0	6^th^ floor
Mycelia sterilia	12	12	0	1^st^, 3^rd^, 4^th^, 5^th^, 6^th^, and 7^th^ floor
Total	48	44	4	

Yeasts				
*Aureobasidium pullulans*	5	4	1	3^rd^,4^th^, and 6^th^ floor
*Candida parapsilosis*	5	4	1	Ground floor, 2^nd^ 3^rd^, and 8^th^ floor
*Candida famata*	2	0	2	3^rd^ floor
*Trichosporon mucoides*	2	2	0	2^nd^ and 5^th^ floor
*Candida intermedia*	1	0	1	3^rd^ floor
*Candida lusitaniae*	1	1	0	7^th^ floor
*Candida membranifaciens*	1	1	0	2^nd^ floor
*Candida globosa*	1	1	0	2^nd^ floor
*Candida pelicullosa*	1	1	0	2^nd^ floor
*Cryptococcus laurentii*	1	0	1	2^nd^ floor
*Rhodotorula minuta*	1	1	0	7^th^ floor
*Sporobolomyces salmonicolor*	1	1	0	5^th^ floor
Total	22	16	6	

*n*: total of samples; *T*: tap water; *P*: purifiers.

## Data Availability

The data used to support the findings of this study are included within the article.
